# Establishing preliminary normative reference values for oral motor skills in typically developing Egyptian children using oral placement therapy tools

**DOI:** 10.1186/s12887-026-07555-2

**Published:** 2026-09-18

**Authors:** Rehab Abd Elhafeez Zaytoun, Aisha Fawzy Abdel Hady, Mohamed Eid Farag, Rasha Abdelrahman Alasheery, Manar Mohsen Gomaa, Sameh Mahmoud Amin

**Affiliations:** 1 Faculty of Medicine, Fayoum university, Fayoum, Egypt; 2 Faculty of Medicine, Cairo university, Cairo, Egypt

**Keywords:** Oral motor assessment, Oral Placement Therapy, Egyptian children, Normative data, Standardization

## Abstract

**Introduction:**

Oral motor skills are essential for normal feeding and speech development. Impairments in these functions may adversely affect a child’s growth, communication abilities, and overall quality of life. Accurate and standardized assessment of oral motor function is therefore critical for early diagnosis and intervention. However, many currently available assessment tools lack adequate objectivity and standardization.

**Aim:**

This study aimed to evaluate oral motor function in typically developing Egyptian children aged 2–6 years using Oral Placement Therapy (OPT) tool hierarchies and to establish normative reference values for clinical use.

**Subjects and methods:**

A cross-sectional study was conducted on 275 typically developing children randomly selected from nurseries in Fayoum, Egypt. Participants were categorized into 6-month age intervals across the 2–6-year age range. Oral motor skills were assessed using OPT tool hierarchies, including bite blocks, bite tubes, horn hierarchies, straw hierarchies, and bubble-blowing tasks.

**Results:**

Oral motor performance demonstrated an overall age-related progression across most assessment domains. Children younger than 42 months consistently achieved lower scores, whereas children older than 66 months achieved the highest scores across most OPT tool hierarchies. Significant differences were observed among age groups in measures of jaw stability, jaw strength, lip function, and oral airflow control.

**Conclusion:**

OPT tool hierarchies provide a feasible and practical method for assessing oral motor function in typically developing Egyptian children. The preliminary reference data generated in this study may serve as a useful clinical guide for the early identification and management of oral motor disorders. However, multi-center validation and comprehensive psychometric evaluation are warranted before these data can be adopted as standardized clinical benchmarks.

## Introduction

Oral motor skills, encompassing the coordinated function of the lips, cheeks, jaw, and tongue, are fundamental components of the stomatognathic system [1]. These abilities are critical for the development of feeding and speech processes in children, significantly impacting their overall growth and well-being [2].

The development of oral motor skills begins in utero and continues through the first two to three years of life, coinciding with the emergence of early language and feeding milestones. While this system responds to tactile stimulation as early as the seventh week of gestation, full refinement of its movements is not achieved until a child reaches six or seven years of age [3].

It promotes healthy growth, facilitates communication, and supports overall development [4]. Conversely, difficulties in these functions can lead to malnutrition and disrupt a child’s physical and social progress, underscoring the vital need for timely intervention [2].

Assessment of oral motor skills ranges from general observation to structured assessments, a significant limitation is that most currently available techniques are highly subjective and lack robust standardization [5].

Among the emerging methods for oral motor skills evaluation is assessment using Oral Placement Therapy (OPT) tools. Developed by Sara Rosenfeld-Johnson, OPT is a specialized component of oral motor therapy that targets the motor elements and movements essential for clear speech production. It distinguishes itself by integrating tactile and proprioceptive sensory input alongside auditory and visual stimuli, allowing children to perceive, hear, and physically experience movements crucial for speech clarity [5]. Critically, OPT provides a structured framework for assessing specific articulatory movements, including awareness, placement, stability, and muscle memory, which are vital for speech development. This systematic approach allows for repeated assessment and the identification of movement deficits through specific tools and tactile-proprioceptive techniques [6].

Unlike many existing oral motor scales that often evaluate global behaviors and rely on subjective terminology [4]. OPT offers distinctive advantages. It focuses on specific individual oral motor movements, with each trial requiring adherence to clear success criteria, thereby minimizing the oversight of subtle changes. Furthermore, OPT provides quantifiable measurement by assessing tasks through a progressive hierarchy of tools, where mastery is determined by repetitions or sustained duration rather than relying on unobservable variables or ambiguous terms like “efficiently” or “coordinated.” This structured, quantifiable approach enhances objectivity and the comparability of results. Moreover, the hierarchical nature of OPT tasks allows for adaptation across various ages and severity levels—which is not possible with many other assessment scales [7]—making it suitable for a broad range of pediatric populations. Recognizing these strengths, establishing preliminary reference range for OPT assessment is critical to provide objective preliminary benchmarks for clinical evaluation.

While we acknowledge that a single-governorate sample limits the generalizability of our findings as definitive national standards, the absence of any previously published OPT-based oral motor data in Egyptian children necessitates the establishment of initial clinical reference anchors. These preliminary benchmarks are intended to provide a structured framework for clinicians, pending future multi-center validation studies.

Given the limitations of current oral motor assessment tools and the promising features of OPT, the importance of standardizing OPT assessment in specific populations, such as typically developing Egyptian children, becomes evident. Such standardization would establish preliminary reference data, providing a much-needed objective reference.

## Subjects and methods

### Subjects

This study was approved by the Medical Research Ethical Committee of the Faculty of Medicine, Fayoum University (M 662). It was a cross-sectional descriptive study conducted between July 2023 and June 2024. The study sample comprised 275 Egyptian children aged between 2 and 6 years.

The participants were divided into 8 subgroups (labeled alphabetically from A to H), each representing a 6-month age interval. This narrow age interval aimed to provide close, focused observation of the children’s oral motor skill development, allowing for the detection of subtle behavioral changes and new milestones over a short timeframe during their early developmental years. The participating children were randomly selected from various nurseries in Fayoum, Egypt.

#### Inclusion criteria

- Typically developing children, as verified by comprehensive history taking and the Ages and Stages Questionnaire (ASQ) Charafeddine et al. (2013) [8]
- Age range between 2 and 6 years

#### Exclusion criteria

- Children presenting with craniofacial abnormalities
- Children with diagnosed neurological disorders
- A history of delayed developmental milestones
- A history of feeding or swallowing disorders

### Study design

- Cross-sectional descriptive study.

### Pilot study

A pilot study involving 32 children from different age groups was conducted prior to the main research. This pilot aimed to assess the children’s capacity to comprehend instructions and perform the various required tasks.

### Methods

All children enrolled in the study underwent a comprehensive evaluation protocol consisting of the following three components:

### Full medical history

A detailed clinical history was taken, including personal, prenatal, natal, and postnatal history, as well as records of previous surgeries or chronic diseases, with a specific focus on:

- *Developmental History*: Mother recognition, social smile, sitting, standing, crawling, walking, and toilet training.
- *Speech History*: Time of onset of babbling, first word, first sentence, and overall speech intelligibility.

### Milestone development assessment (ASQ)

Milestone development was assessed using the Arabic version of the Ages and Stages Questionnaires (ASQ), translated by Charafeddine et al. (2013) [8]. The questionnaire was completed by the children’s parents. Only children whose scores fell within the normal range for developmental milestones were included in the study.

The ASQ is a standardized, age-tailored screening tool covering five primary areas of child development: communication, gross motor, fine motor, problem-solving, and personal-social skills. It has been demonstrated to be a dependable, economical instrument with a strong correlation to professional medical evaluations [9].

### Oral motor assessment by OPT tools

Oral motor function was evaluated using five tools from Oral Placement Therapy (OPT):

1. Bite Blocks
2. Bite Tubes
3. Straw Hierarchy
4. Horn Hierarchy
5. Bubble-Blowing Hierarchy

### General administration protocol

- Before assessing each task, the examiner demonstrated how to perform the task clearly in front of the child.
- The child was given 3 trials per task, and the average score was estimated.
- If a compensatory error occurred, the trial was discontinued immediately.
- During testing, the child sat on a suitable chair with their back and feet fully supported.
- The examiner scored each task in real-time based on the predefined success criteria and compensatory error checklists.

### Specific OPT assessment hierarchies

A. Jaw grading bite blocks

This hierarchy utilizes 6 bite blocks graded from block number 2 to 7. This task is designed to assess jaw stability,jaw strength, and graded jaw grading capacity, which are foundational for sustained biting, chewing, and articulatory precision. Each bite block is used for 3 specific exercises (A, B, and C). To participate, the child must possess the cognitive ability and motor skills required for the prerequisite “bite and hold” exercise without exhibiting compensatory movements [10].

These exercises were applied to children aged 3 years and older. In our pilot study, children under 3 years old consistently failed to perform the exercises, primarily due to an inability to comprehend the requested maneuvers rather than a true lack of oral motor capability. Consequently, accurate results could not be obtained for this younger age group. Additionally, in most children, the eruption of the upper and lower primary back molars is completed during the third year of life [11].

- *Prerequisite*: Optimal bite posture, fully erupted upper and lower back molars, and comprehension of the “bite and hold” concept.
- *Criteria for Success*:
  - *Exercise A (Bite Block Exercise)*: The bite block is placed on the surface of both back molars, extending from the front of the mouth. The child must hold a natural bite posture on each block for 15 s while maintaining an isometric pull on the left and then the right side, without any evidence of compensatory patterns. This exercise assesses lateral jaw stability and the ability to maintain a steady bite against resistance.
  - *Exercise B (Twin Bite Block Exercise)*: Two bite blocks are placed on the surfaces of both back molars simultaneously. The child must maintain a natural bite posture on each block for 15 s while isometric pull is applied to both sides at the same time, without evidence of compensatory patterns. This exercise assesses bilateral jaw stability and symmetric bite force distribution.
  - *Exercise C (Bite Block for Jaw Stability Exercise)*: A bite block is placed on the surface of the lower teeth extending across the midline. The child must maintain the bite and pull forward against isometric resistance. The examiner holds the isometric pull for 15 s. This exercise assesses anterior-posterior jaw stability and resistance to forward displacement.

In each exercise, the examiner counted to 15 or until the child shows compensatory patterns or released the block. If the child successfully completes the criteria in one exercise, the examiner progresses to the next exercise.

- *Compensatory Patterns to Avoid*: Head movement, jaw sliding, or jaw jutting.
- *Scoring*: Based on the highest bite block number achieved (maximum 7) and the duration of the exercise sustained (maximum 15 s).

#### Standardization of isometric pull

To ensure consistency across participants, the examiner applied a gentle, sustained isometric pull using a standardized hand grip technique. The force was applied in a steady horizontal direction—laterally for Exercises A and B, and anteriorly for Exercise C—and was maintained continuously throughout each 15-second trial. While formal force measurement (e.g., using a dynamometer) was not employed, the examiner received standardized training under the supervision of senior phoniatricians to ensure consistent application of pull force and duration across all participants. The use of a single trained examiner throughout the study further minimized variability in force application. This procedural standardization is important for reproducibility and for clinicians wishing to adopt this assessment protocol.

B. Bite tube hierarchy

This hierarchy utilizes 4 Chewy Tubes color-coded by level: red (lowest level), followed by yellow, purple, and green (highest level). This task is designed to assess jaw strength, jaw grading, and repetitive chewing endurance, which are essential for functional chewing and sustained oral motor activity. Each tube was placed on the surface of the lower back molars, extending from the side of the mouth. The examiner instructed the child to chew with complete compression on the tube, counting the number of sequential chews completed without compensatory errors.

- *Criteria for Success*: The child must bite the tube 10 times on each side without compensatory posturing to complete a given bite tube level. The success criterion for progressing to the next tube is the ability to bite the tube 2 times on each side without compensatory posture¹¹.
- *Compensatory Patterns to Avoid*: Jaw sliding or jaw jutting.
- *Scoring*: Based on the bite tube level reached (maximum number 4) and the number of repetitions completed (maximum 10 repetitions).

C. Horn blowing hierarchy

This hierarchy consists of 12 horns that gradually increase in difficulty, with horn number 1 being the easiest and horn number 12 being the most difficult. This task is designed to assess lip closure, lip rounding, tongue retraction, respiratory support, and sustained oral airflow control, which are critical for speech production, articulation precision, and intraoral pressure generation. The examiner held the horn perpendicular to the child’s mouth and parallel to the floor, resting it on the surface of the child’s lower lip at the midline. The child is then asked to take a breath in and blow.

- *Criteria for Success*: Each horn must be blown 25 times in rapid succession utilizing only lip and abdominal grading, with no compensatory posturing. The child must achieve the target duration criteria for each horn to proceed to the next:
  - *Horn 1*: Any duration
  - *Horn 2*: 1 s
  - *Horns 3 & 4*: More than 1 s
  - *Horns 5*,* 6*,* & 7*: 2 s
  - *Horns 8 & 9*: More than 2 s
  - *Horns 10*,* 11*,* & 12*: 3 s
- *Compensatory Errors to Avoid*: Teeth biting on the horn tip, body extensor patterns (e.g., shoulder elevation), and lip retraction.
- *Scoring*: Based on the horn number achieved (maximum 12) and the number of repetitions completed (maximum 25 repetitions).

D. Straw hierarchy

This hierarchy consists of 8 straws that gradually increase in difficulty. Straw number 1 is the easiest and straw number 8 is the most difficult. The examiner began the assessment using either straw number 1 or straw number 4. This task is designed to assess lip protrusion, jaw-lip dissociation, tongue retraction, intraoral pressure generation, and coordinated swallowing, which are essential for efficient feeding, drinking, and articulatory precision.

- *Criteria for Success (Straws 1, 2, & 3)*: The child must successfully drink 3 ounces (88.7 ml) of any thin liquid through the straw with protruded lips and a retracted tongue. Either single sips or repetitive continuous draws are acceptable.
- *Criteria for Success (Straws 4 through 8)*: The child must use a single-sip technique adhering to the following sequence: (a) place the straw between the lips, (b) draw the liquid until it is felt in the mouth, (c) remove the straw, (d) close the lips, and (e) swallow. A total of 10 sips must be taken using this technique without compensatory errors.
- *Compensatory Errors to Avoid*: Lip retraction, body extensor patterns (e.g., shoulder elevation), and teeth biting on the straw that inhibits jaw-lip dissociation.
- *Scoring*: Based on the straw number reached (maximum 8) and the number of repetitions completed (maximum 10 repetitions).

E. Bubble blowing hierarchy

The examiner held the bubble wand approximately 1 inch away from the child’s mouth. This task is designed to assess jaw stability, lip rounding, tongue retraction, respiratory support, and coordinated oral airflow, which are foundational for speech production and oral motor coordination. Assessment is conducted using a two-step sequence:

- *Step 1 (Blowing the bubble off the wand)*: The child blows air directly at the bubble solution on the wand to push the bubble off the wand and into the air. The bubble is formed by the examiner’s dipping of the wand; the child’s role is to generate sufficient airflow to detach the bubble from the wand surface. This step assesses basic oral airflow generation and lip approximation.
- *Step 2 (Blowing the bubble through the wand)*: The child blows air through the wand’s circular ring, with the bubble solution forming a film across the ring, to create the bubble from the solution on the wand and then blow it through the ring into the air. This requires the child to produce a more sustained and directed stream of air to actually form the bubble, demanding greater sustained, directed oral airflow and coordinated lip rounding.

The key distinction is that Step 1 requires only sufficient airflow to detach a pre-formed bubble, whereas Step 2 requires the child to generate the bubble themselves by blowing through the film of solution, which demands greater respiratory support, lip rounding precision, and sustained airflow control.

- *Criteria for Success*: Each step must be completed 10 times in rapid repetition without a break or compensatory errors before moving to the next step.
- *Compensatory Errors to Avoid*: Shoulder elevation, whole-body extension, high or low jaw fixing.
- *Scoring*: Based on the step reached (highest through-wand performance) and the number of repetitions completed (maximum 10 repetitions).

### Psychometric properties of the OPT assessment tools

The OPT assessment tools used in this study were developed based on established theoretical frameworks of oral motor development and have been previously validated in clinical populations through extensive clinical application (Rosenfeld-Johnson, 2009). Content validity of the OPT hierarchies was ensured through the systematic selection of tasks that target specific, developmentally sequenced oral motor components, including jaw stability (bite blocks), jaw strength and chewing patterns (bite tubes), lip rounding and respiratory control (horn hierarchy), lip protrusion and intraoral pressure generation (straw hierarchy), and coordinated airflow control (bubble-blowing). Each task within the hierarchies was designed to assess discrete oral motor functions with clearly defined success criteria, thereby minimizing subjective interpretation.

Construct validity was supported by the hierarchical progression of each tool, which was designed to reflect the developmental continuum of oral motor skill acquisition. The tools were selected to assess distinct but interrelated domains of oral motor function, including jaw stability, lip function, tongue retraction, and respiratory support, which are theoretically and clinically recognized as essential components of feeding and speech development (Sampallo-Pedroza et al., 2014).

It is important to note that the present study was designed to generate preliminary normative reference data for clinical guidance rather than to establish the OPT tools as a fully standardized psychometric instrument. While the assessment tasks were developed based on established theoretical frameworks and include structured, objective success criteria that support content and construct validity, comprehensive standardization—including inter-rater and intra-rater reliability, test-retest reliability, and concurrent validity—would be required before the tools could be considered a fully standardized instrument. The current findings represent an initial step toward this goal. 

### Assessor information and reliability

All assessments were performed by a single trained examiner (MMG), a certified speech-language pathologist with specialized training in Oral Placement Therapy, to ensure consistency in administration and scoring. The examiner underwent standardized training in the use of OPT assessment tools under the supervision of senior phoniatrician (RAZ), who is expert in the field of oral motor assessment.

Although formal inter-rater and intra-rater reliability coefficients were not calculated in the present study due to the single-examiner design, the assessment protocol was highly standardized, with each task requiring adherence to objective, predefined success criteria (e.g., number of repetitions, sustained duration in seconds, specific levels achieved). This structured, criteria-based approach inherently reduces scorer variability compared to subjective rating scales. Future studies should incorporate independent inter-rater and intra-rater reliability assessments to further strengthen the psychometric evidence base of the OPT tools. This limitation is acknowledged in the limitations section of the manuscript.

### Statistical methods

- Data Management: Collected data were coded to facilitate manipulation, double-entered into Microsoft Access, and statistically analyzed using the Statistical Package for the Social Sciences (SPSS) software version 22 for Windows 7 (SPSS Inc., Chicago, IL, USA).
- Descriptive Statistics: Qualitative data were analyzed using numbers and percentages. For quantitative data, arithmetic means and standard deviations were used for normally distributed variables, while medians and ranges were reported for non-normally distributed variables.
- Normality Testing: Quantitative data were evaluated for normality within each study group using the One-Sample Kolmogorov-Smirnov test. The results guided the selection of parametric versus non-parametric tests. Variables demonstrating normal distribution (bite block number scores and straw hierarchy number and repetition scores) were analyzed using parametric tests. Variables with non-normal distribution (bite tube repetition scores, bite block duration scores in younger groups, and bubble blowing scores) were analyzed using non-parametric tests. Horn hierarchy scores showed mixed normality patterns, and non-parametric tests were applied conservatively. The ‘Expected Ranges’ presented in the tables (calculated as Mean ± 1 SD) are provided for descriptive purposes to illustrate the central distribution and variability of scores within each age subgroup. They are not intended to serve as formal clinical reference intervals or diagnostic cut-offs, which typically require 95% coverage (Mean ± 1.96 SD) and larger, multi-center normative samples (CLSI guidelines).
- *Inferential Statistics*:
- Parametric tests: One-way ANOVA was used to compare quantitative measures among age groups. Post-hoc pairwise comparisons were performed using Tukey’s Honestly Significant Difference (HSD) test.
- Non-parametric tests: Kruskal-Wallis test was used to compare quantitative measures among age groups. Post-hoc pairwise comparisons were performed using Mann-Whitney U tests with Bonferroni correction.
- For qualitative data: Chi-square test was employed to compare exercise completion rates among age groups.
- Effect Sizes: To quantify the magnitude of observed differences, effect sizes were calculated for all statistically significant comparisons. For parametric tests, partial eta squared (η²p) was reported for ANOVA, and Cohen’s d was calculated for pairwise comparisons. For non-parametric tests, epsilon-squared (ε²) was reported for Kruskal-Wallis tests, and r = Z/√N was calculated for Mann-Whitney pairwise comparisons. Effect sizes were interpreted according to conventional criteria: partial eta squared (small = 0.01, medium = 0.06, large = 0.14); Cohen’s d (small = 0.2, medium = 0.5, large = 0.8); ε² (small = 0.01, medium = 0.06, large = 0.14); r (small = 0.1, medium = 0.3, large = 0.5).
- Significance Level: A *P*-value < 0.05 was considered statistically significant.

## Results

Tables 1 showed that the age range included in the current study was between 24 and 72 months. Males constituted about 52.7% while females constituted 47.3% of the study group.

**Table 1:** Demographic characteristics of the study group

Variables (*n* = 275(	Frequency	
	No.	%
Age groups		
Group (A) 24- <30 m	30	10.9%
Group (B) 30- <36 m	38	13.8%
Group (C) 36- <42 m	42	15.3%
Group (D) 42- <48 m	34	12.4%
Group (E) 48- <54 m	37	13.5%
Group (F) 54- <60 m	31	11.3%
Group (G) 60- <66 m	32	11.6%
Group (H) 66- <72 m	31	11.3%
Sex		
Male	145	52.7%
Female	130	47.3%

Table 2 showed that bite block performance generally improved with age. Mean bite block level increased from 2.83 ± 0.85 in Group C to 6.42 ± 0.85 in Group H (*p* < 0.001). Although the overall trend showed age-related improvement, a small, non-significant decrease was observed between Group D (42–<48 months) and Group E (48–<54 months), followed by a sharp increase in Group F (54–<60 months). Given the cross-sectional design, this isolated fluctuation is most parsimoniously attributed to individual variability within the sample rather than a genuine developmental regression. The general pattern across the entire age range nevertheless supports progressive maturation of jaw stability and strength.

**Table 2:** Jaw grading bite blocks – preliminary reference values by age group

Age Group	*n*	Mean Block Level ± SD	Range	Expected Range (Mean ± 1 SD)	Mean Duration (sec) ± SD	Expected Duration Range (Mean ± 1 SD)	Exercise		
							A	B	C
Group (C) 36- <42 m	42	2.83 ± 0.85	1–4	1.98–3.68	8.1 ± 3.7	4.4–11.8	41(97.6%)	1(2.4%)	0(0%)
Group (D) 42- <48 m	34	4.27 ± 1.13	2–7	3.14–5.40	9.6 ± 3.4	6.2–13.0	28(82.4%)	5(14.7%)	1(2.9%)
Group (E) 48- <54 m	37	3.70 ± 0.81	2–6	2.89–4.51	9.5 ± 3.3	6.2–12.8	33(89.2%)	2(5.4%)	2(5.4%)
Group (F) 54- <60 m	31	5.81 ± 0.91	3–7	4.90–6.72	9.4 ± 3.3	6.1–12.7	20(64.5%)	6(19.4%)	5(16.1%)
Group (G) 60- <66 m	32	5.59 ± 0.79	4–7	4.80–6.38	9.8 ± 3.9	5.9–13.7	28(87.5%)	2(6.3%)	2(6.3%)
Group (H) 66- <72 m	31	6.42 ± 0.85	5–7	5.57–7.27	11.6 ± 3.1	8.5–14.7	14(45.2%)	6(19.4%)	11(35.5%)

Duration of bite maintenance also increased with age overall, with significant differences on both right (*p* < 0.001) and left (*p* = 0.005) sides. However, similar to the block level scores, duration showed slight fluctuations between adjacent age groups rather than a strictly linear progression. Exercise completion showed progressive improvement overall: Exercise A was achieved by 97.6% of Group C but only 45.2% of Group H, while Exercise C was completed by 0% of Group C versus 35.5% of Group H (*p* < 0.001).

Performance on the bite tube hierarchy demonstrated significant age-related improvement across all four tube levels and both sides (*p* < 0.001 for all comparisons). For the red tube, mean repetition scores increased from 1.9 ± 1.8 in Group A to 8.6 ± 1.8 in Group H. Yellow tube scores increased from 1.5 ± 1.6 in Group A to 8.3 ± 1.9 in Group H. Purple tube scores increased from near-zero levels in Group A to 5.3 ± 2.8 (right) and 5.8 ± 2.7 (left) in Group H. The green tube, which was not achieved by any child in Group A, showed mean scores of 3.4 ± 2.5 (right) and 3.3 ± 2.5 (left) in Group H. Side-to-side differences were minimal across all age groups, suggesting symmetrical development of jaw strength and chewing endurance. 

Expected range scores, presented in Table 4, confirm the progressive pattern observed in Table 3. Children in Group H achieved median scores of 10 for the red tube, 9–10 for the yellow tube, 5–6 for the purple tube, and 3 for the green tube. The ranges reveal substantial individual variability within each age group, particularly for higher difficulty levels. This variability may reflect individual differences in maturation, prior exposure, or task comprehension. The progressive increase in median scores with age, with narrowing ranges in older groups, suggests that jaw strength and chewing endurance become more consistently developed as children mature. 

Horn levels achieved increased significantly with age, from 1.2 ± 0.40 in Group A to 4.2 ± 0.88 in Group H (*p* < 0.001), reflecting overall maturation of lip closure, rounding, tongue retraction, and respiratory support. The progression was generally consistent, with steady increases across most age intervals.

In contrast, repetition scores showed no significant age-related trend (*p* = 0.23) and demonstrated marked fluctuation across adjacent age groups. The highest mean repetitions were unexpectedly observed in Group B (14.2 ± 10.2), followed by Group A (12.4 ± 7.8), with scores subsequently decreasing in Group C (8.0 ± 9.1) and remaining relatively stable across older groups (ranging from 8.3 ± 4.9 to 9.02 ± 5.6). This non-significant and fluctuating pattern suggests that as the level of the horn progress the difficulty increase and the ability to repeat for multiple times decrease. 

Straw performance demonstrated generally progressive improvement with age. Mean straw level increased from 2.4 ± 0.77 in Group A to 7.7 ± 0.69 in Group H (*p* < 0.001). Repetition scores also improved significantly overall (*p* < 0.001), increasing from 5.7 ± 2.9 in Group C to 9.1 ± 1.9 in Group H. However, repetition scores showed a slight decrease between Group D (5.9 ± 2.3) and Group E (4.9 ± 1.9), followed by a steady increase through Group H (9.1 ± 1.9). Given the cross-sectional nature of the data, this minor fluctuation is likely attributable to sampling variability or individual challenges encountered during the transition to more advanced straw levels requiring the single-sip technique rather than a meaningful developmental phenomenon. The overall trend nevertheless indicates progressive improvement in straw-drinking proficiency across the 2–6-year age range.

Younger children frequently demonstrated difficulty with lip protrusion and jaw-lip dissociation, often stabilizing the straw between their teeth. The generally progressive pattern observed suggests steady maturation of lip strength and intraoral pressure generation, with some intermittent variability across adjacent age groups. 

Bubble blowing performance demonstrated overall significant improvement across both steps (*p* < 0.001). Step 1 (off wand) scores increased from 6.3 ± 2.9 in Group A to 10 ± 0 in Groups G and H, with mastery achieved by approximately 48 months. Step 2 (through wand) scores increased from 0.83 ± 1.6 in Group A to 10 ± 0 in Groups G and H. However, Step 1 scores showed a small decrease between Group B (8.8 ± 2.5) and Group C (7.5 ± 3.04), followed by continued improvement through Group H. In the absence of longitudinal data, this isolated fluctuation is best interpreted as random sampling variation or differing levels of prior task familiarity among participants, rather than a true developmental pattern. The overall progressive improvement across age groups supports the utility of bubble-blowing as a marker of oral airflow maturation.

Step 2 scores showed a more consistent progressive pattern, with notable improvement beginning in Group D (42–48 months; mean 6.5 ± 4.2) and reaching mastery in Groups G and H. Younger children frequently exhibited cheek puffing, reflecting immature airflow control and lip rounding. The progressive improvement across age groups reflects the ongoing development of coordinated respiratory support and oral airflow regulation.

## Discussion

The identification of specific oral-motor skill deficits is essential for the diagnosis and management of feeding and speech disorders. Despite the availability of several oral motor assessment methods, the lack of standardized procedures, limited normative data, and reliance on subjective clinical observations remain significant challenges. These limitations hinder comparisons across studies, clinics and assessment periods [4]. Therefore, the present study aimed to evaluate oral motor skills using Oral Placement Therapy tools in typically developing Egyptian children and to establish normal reference values for clinical application.

Throughout this discussion, improvements in oral motor performance are interpreted as potentially reflecting maturation of underlying physiological functions, including jaw strength, lip rounding, tongue retraction, respiratory support, abdominal muscle control, and intraoral pressure generation. However, it is important to emphasize that these physiological parameters were not directly measured in the present study. The OPT assessment tasks provide indirect behavioral indicators of these functions rather than direct physiological measurements. Therefore, all interpretations linking performance to specific physiological mechanisms should be viewed as hypothesis-generating rather than conclusive. Future studies incorporating direct physiological measures (e.g., manometry for intraoral pressure, electromyography for muscle activity, or spirometry for respiratory function) would be valuable to confirm the mechanisms underlying the observed behavioral improvements.

The present findings demonstrate an overall age-related improvement in oral motor performance across the 2–6-year age range. However, as this was a cross-sectional study, the observed fluctuations and minor variations between adjacent age groups should be interpreted with caution. Such variations may reflect normal individual differences, sampling variability, task comprehension, or attentional factors rather than true developmental regression or nonlinear growth patterns. Longitudinal studies are needed to confirm whether these fluctuations represent genuine developmental phenomena or merely statistical noise arising from cross-sectional sampling.

Regarding jaw grading using bite blocks, performance improved significantly with age. Both the level of bite block achieved and the duration of task performance increased across age groups, which may reflect progressive development of jaw strength and stability. Children younger than 36 months were not assessed using bite blocks because pilot observations indicated that they were generally unable to understand or perform the task reliably. This finding is consistent with previous reports [7, 12] indicating that oral motor assessments may place substantial cognitive demands on young children, potentially affecting task performance independent of actual motor ability. The most frequently observed compensatory patterns were jaw sliding and lateralization, particularly among younger children with less developed jaw stability.

Performance on the bite tube hierarchy as shown in Tables (3 and 4) also demonstrated a significant age-related improvement. Children younger than 42 months showed lower repetition scores, likely reflecting immature jaw strength, motor control, and task comprehension. In contrast, children older than 66 months achieved the highest performance levels. Younger children frequently demonstrated compensatory jaw movements, including sliding and lateralization. These findings are consistent with the developmental progression of chewing patterns described in the literature [13], in which mandibular movements evolve from simple vertical movements to more complex rotary chewing patterns as children mature.

**Table 3:** Comparisons of the mean number of repetitions of bite tubes in different age groups

Age groups	Bite tube							
	Red		Yellow		Purple		Green	
	Right	Left	Right	Left	Right	Left	Right	Left
	Mean ± SD	Mean ± SD	Mean ± SD	Mean ± SD	Mean ± SD	Mean ± SD	Mean ± SD	Mean ± SD
Group (A) 24- <30 m	1.9 ± 1.8	1.9 ± 1.8	1.5 ± 1.6	1.5 ± 1.8	0.1 ± 0.31	0.07 ± 0.25	0 ± 0	0 ± 0
Group (B) 30- <36 m	3.4 ± 2.6	3.9 ± 2.9	3.1 ± 2.5	3.2 ± 2.8	0.73 ± 1.3	0.97 ± 1.8	0.26 ± 0.76	0.37 ± 0.91
Group (C) 36- <42 m	3.9 ± 2.7	4.1 ± 2.9	3.2 ± 2.7	3.5 ± 3.1	0.69 ± 1.3	0.83 ± 1.8	0.19 ± 0.39	0.24 ± 0.53
Group (D) 42- <48 m	5.7 ± 2.6	5.9 ± 2.6	5.5 ± 2.7	5.9 ± 2.7	2.4 ± 2.4	2.3 ± 2.1	1.1 ± 1.8	0.88 ± 0.13
Group (E) 48- <54 m	5.8 ± 2.6	6.2 ± 2.7	5.4 ± 2.6	5.6 ± 2.8	2.4 ± 2.2	2.5 ± 2.5	0.97 ± 1.3	0.95 ± 1.4
Group (F) 54- <60 m	7.7 ± 1.9	8.1 ± 1.9	7.6 ± 1.9	7.9 ± 1.8	3.7 ± 2.3	4.1 ± 2.3	1.7 ± 1.5	1.9 ± 1.4
Group (G) 60- <66 m	7.2 ± 1.8	7.4 ± 1.7	7 ± 1.7	6.8 ± 2	3.6 ± 1.9	3.6 ± 2.1	1.8 ± 1.9	1.7 ± 2.2
Group (H) 66- <72 m	8.6 ± 1.8	8.7 ± 1.7	8.3 ± 1.9	8.7 ± 1.7	5.3 ± 2.8	5.8 ± 2.7	3.4 ± 2.5	3.3 ± 2.5
*P*-value	**< **0.001	**< **0.001	**< **0.001	**<** 0.001	**< **0.001	**< **0.001	**< **0.001	**<** 0.001
Sig.	HS	HS	HS	HS	HS	HS	HS	HS

**Table 4:** Bite tube hierarchy – expected ranges (Mean ± 1 SD) by age group (right and left sides)

Age groups	*n*	Bite tube Expected Ranges							
		Red		Yellow		Purple		Green	
		Right	Left	Right	Left	Right	Left	Right	Left
Group (A) 24- <30 m	30	0.1–3.7	0.1–3.7	0.0–3.1	0.0–3.3	0.0–0.41	0.0–0.32	0.0–0.0	0.0–0.0
Group (B) 30- <36 m	38	0.8–6.0	1.0–6.8	0.6–5.6	0.4–6.0	0.0–2.03	0.0–2.77	0.0–1.02	0.0–1.28
Group (C) 36- <42 m	42	1.2–6.6	1.2–7.0	0.5–5.9	0.4–6.6	0.0–1.99	0.0–2.63	0.0–0.58	0.0–0.77
Group (D) 42- <48 m	34	3.1–8.3	3.3–8.5	2.8–8.2	3.2–8.6	0.0–4.8	0.2–4.4	0.0–2.9	0.75–1.01
Group (E) 48- <54 m	37	3.2–8.4	3.5–8.9	2.8–8.0	2.8–8.4	0.2–4.6	0.0–5.0	0.0–2.27	0.0–2.35
Group (F) 54- <60 m	31	5.8–9.6	6.2–10.0	5.7–9.5	6.1–9.7	1.4–6.0	1.8–6.4	0.2–3.2	0.5–3.3
Group (G) 60- <66 m	32	5.4–9.0	5.7–9.1	5.3–8.7	4.8–8.8	1.7–5.5	1.5–5.7	0.0–3.7	0.0–3.9
Group (H) 66- <72 m	31	6.8–10.4	7.0–10.4	6.4–10.2	7.0–10.4	2.5–8.1	3.1–8.5	0.9–5.9	0.8–5.8

Assessment using the horn hierarchy (Table 5) revealed progressive improvements with increasing age. Higher horn levels and greater repetition scores were observed among older children, which may reflect maturation of lip closure, lip rounding, tongue retraction, respiratory support, and oral airflow control. It is important to note that respiratory support and muscle function were not directly assessed in this study; therefore, these explanations should be considered as possible mechanisms rather than established conclusions. The most common difficulty observed among younger participants was failure to sustain the airflow duration required for higher-level horns. This finding may be related to limited abdominal muscle control and reduced ability to regulate expiratory airflow, both of which are important for efficient speech production and oral motor performance [14].

**Table 5:** Horn blowing hierarchy – preliminary reference values by age group

Age Group	*n*	Mean Horn Level ± SD	Range	Expected Range (Mean ± 1 SD)	Mean Repetitions ± SD	Expected Repetition Range (Mean ± 1 SD)
Group (A) 24- <30 m	30	1.2 ± 0.40	1–2	0.8–1.6	12.4 ± 7.8	4.6–20.2
Group (B) 30- <36 m	38	1.4 ± 0.60	1–3	0.8–2.0	14.2 ± 10.2	4.0–24.4
Group (C) 36- <42 m	42	1.6 ± 0.56	1–3	1.04–2.16	8.0 ± 9.1	0.0–17.1
Group (D) 42- <48 m	34	2.6 ± 0.82	1–5	1.78–3.42	9.02 ± 5.6	3.42–14.62
Group (E) 48- <54 m	37	2.8 ± 0.86	2–5	1.94–3.66	8.4 ± 5.1	3.3–13.5
Group (F) 54- <60 m	31	3.7 ± 0.83	2–5	2.87–4.53	8.3 ± 4.9	3.4–13.2
Group (G) 60- <66 m	32	3.6 ± 0.74	3–6	2.86–4.34	8.7 ± 4.9	3.8–13.6
Group (H) 66- <72 m	31	4.2 ± 0.88	3–7	3.32–5.08	8.5 ± 5.4	3.1–13.9

Similarly, performance on the straw hierarchy (Table 6) improved significantly across age groups. Younger children often demonstrated difficulty maintaining appropriate lip protrusion and jaw-lip dissociation, particularly when using the more advanced straws requiring the single-sip technique. In addition, some children compensated by stabilizing the straw between their teeth rather than using adequate lip control. These findings may reflect the ongoing development of oral motor coordination, lip strength, and intraoral pressure generation, although these parameters were not directly measured in the present study.“. The observed developmental progression is consistent with the established maturation of feeding skills during early childhood. The first time the straw can be used may vary. It can be found between the ages of seven and eight months, when the tongue begins to move more deliberately forward and backward [12]. Other opinion said by Reilly [13] that the age of 2 years is the average age when children are able to drink from a straw. However, some children as young as 6 months can drink from a straw the experience is very important regarding this skill. As drinking through a straw is the sole feeding activity that is done against gravity, it is crucial to have adequate intraoral pressure, which demands good lip closure and protrusion [1].

**Table 6:** Straw hierarchy – preliminary reference values by age group

Age Group	*n*	Mean Straw Level ± SD	Range	Expected Range (Mean ± 1 SD)	Mean Repetitions ± SD	Expected Repetition Range (Mean ± 1 SD)
Group (A) 24- <30 m	30	2.4 ± 0.77	1–4	1.63–3.17	—	—
Group (B) 30- <36 m	38	2.8 ± 0.69	1–4	2.11–3.49	—	—
Group (C) 36- <42 m	42	4.04 ± 0.98	2–7	3.06–5.02	5.7 ± 2.9	2.8–8.6
Group (D) 42- <48 m	34	5.02 ± 1.2	3–8	3.82–6.22	5.9 ± 2.3	3.6–8.2
Group (E) 48- <54 m	37	5.7 ± 1.0	4–8	4.7–6.7	4.9 ± 1.9	3.0–6.8
Group (F) 54- <60 m	31	7.0 ± 0.93	5–8	6.07–7.93	7.6 ± 2.5	5.1–10.1
Group (G) 60- <66 m	32	7.1 ± 0.86	6–8	6.24–7.96	7.8 ± 2.6	5.2–10.4
Group (H) 66- <72 m	31	7.7 ± 0.69	2–8	7.01–8.39	9.1 ± 1.9	7.2–11.0

Bubble-blowing performance also demonstrated significant age-related improvement (Table 7). Both the number of successful repetitions and the ability to blow through the wand increased progressively with age. Successful performance requires coordinated jaw stability, lip rounding, tongue retraction, respiratory control, and abdominal muscle activation. Younger children frequently exhibited cheek puffing, suggesting inadequate oral pressure regulation and immature airflow control. Although limited literature is available regarding the use of bubble-blowing tasks as formal assessment measures, the current findings suggest that this task may provide valuable information regarding oral airflow control and respiratory support during oral motor evaluation.

**Table 7:** Bubble blowing hierarchy – preliminary reference values by age

Age groups	Bubble Blowing			
	Off wand		Through wand	
	Mean ± SD	Median (Range)	Mean ± SD	Median (Range)
Group (A) 24- <30 m	6.3 ± 2.9	5(2–10)	0.83 ± 1.6	0(0–5)
Group (B) 30- <36 m	8.8 ± 2.5	10(2–10)	4.9 ± 4.5	4(0–10)
Group (C) 36- <42 m	7.5 ± 3.04	9(1–10)	3.2 ± 3.9	0(0–10)
Group (D) 42- <48 m	8.9 ± 2.3	10(2–10)	6.5 ± 4.2	10(0–10)
Group (E) 48- <54 m	10 ± 0	10(10–10)	6.9 ± 2.9	7(1–10)
Group (F) 54- <60 m	10 ± 0	10(10–10)	9.4 ± 1.6	10(3–10)
Group (G) 60- <66 m	10 ± 0	10(10–10)	10 ± 0	10(10–10)
Group (H) 66- <72 m	10 ± 0	10(10–10)	10 ± 0	10(10–10)
*P*-value	**< **0.001		**< **0.001	
Sig.	HS		HS	

The preliminary normative values generated in this study may serve as a useful clinical guide for practitioners assessing Egyptian children aged 2–6 years. However, given the absence of formal inter-rater reliability data and the limited psychometric evaluation conducted to date, the OPT assessment tool should currently be regarded as a clinical reference framework rather than a fully standardized instrument. Future research should prioritize inter-rater and intra-rater reliability testing, test-retest stability, and concurrent validity with established oral motor assessment scales to support full standardization. Multicenter studies across diverse Egyptian populations are also needed to enhance generalizability. Until such evidence is available, clinicians are encouraged to use these normative values alongside comprehensive clinical history and judgment.

## Conclusion

Several limitations should be acknowledged. First, the study was conducted in a single governorate, which may limit the generalizability of the findings to the broader Egyptian population. Second, the cross-sectional design precluded longitudinal monitoring of individual developmental trajectories. Third, although the assessment protocol was standardized and all evaluations were performed by a single trained examiner to ensure consistency, formal inter-rater and intra-rater reliability coefficients were not calculated. Fourth, while improved performance with age is interpreted as potentially reflecting maturation of underlying physiological mechanisms such as jaw strength, respiratory support, and lip function, these parameters were not directly measured. The OPT tasks provide indirect behavioral indicators of oral motor function rather than direct physiological assessments. Future studies incorporating direct physiological measures would strengthen the evidence base for these interpretations. Future studies should include multiple independent examiners to establish inter-rater reliability and should assess intra-rater reliability through repeated scoring of recorded assessments. Additionally, psychometric properties such as test-retest reliability and concurrent validity with established oral motor assessment tools were not examined and warrant further investigation.

Finally, the cross-sectional design inherently limits our ability to infer true developmental trajectories. While we observed overall age-related improvements, the minor fluctuations between adjacent age groups may reflect cohort effects, sampling variability, or non-motor factors (e.g., attention, motivation, comprehension) rather than genuine developmental patterns. Longitudinal studies are essential to validate these observed trends and to distinguish between maturational change and cross-sectional sampling error.

Furthermore, the descriptive ranges (Mean ± 1 SD) presented in this study should not be mistaken for formal clinical reference intervals. Future large-scale multi-center studies are required to establish definitive 95% reference ranges in accordance with international biostatistical standards.

In conclusion, while this study provides useful preliminary reference values for oral motor skills in typically developing Egyptian children aged 2–6 years using OPT tools, these findings must be interpreted with caution. They currently serve as provisional clinical anchors rather than definitive normative standards. Multi-center studies involving larger and more geographically diverse Egyptian populations are urgently needed to validate these initial reference ranges. Furthermore, comprehensive psychometric evaluation—including inter-rater and intra-rater reliability, test-retest stability, and construct validity—is essential before the OPT tool can be recommended as a fully standardized clinical instrument. Until such evidence is available, clinicians are advised to use these preliminary data alongside comprehensive clinical history and judgment.

## Data Availability

The datasets used and/or analyzed during the current study are available from the corresponding author upon reasonable request. The data are not publicly available due to ethical and privacy restrictions, as they contain sensitive information about participating children.

## Abbreviations

OPT: Oral Placement Therapy
ASQ: Ages and Stages Questionnaire
SPSS: Statistical Package for the Social Sciences
